# PD39 Health Technology Assessment Processes In Brazil: A Comparative Study Of Public And Private Regulatory Frameworks

**DOI:** 10.1017/S0266462325101724

**Published:** 2025-12-29

**Authors:** Ávila Vidal, Ivan Zimmermann

## Abstract

**Introduction:**

In Brazil, national recommendations on health technology assessment (HTA) are carried out differently in the public and private healthcare domains by the National Supplementary Health Agency (ANS) and the National Committee for Health Technology Incorporation (CONITEC), respectively. Nevertheless, since 2018, the ANS officially began aligning its HTA process with the CONITEC process.

**Methods:**

This research employed a qualitative methodology to analyze the ANS and CONITEC HTA processes. Key documents, including laws, guidelines, and policy papers, were gathered from official websites of Brazilian government institutions and national legislation relating to the incorporation process in the period from 2018 to 2024. These texts were subjected to a thematic analysis to identify recurring patterns, discrepancies, and regulatory philosophies. The aim was to compare how different frameworks address common issues such as compliance, enforcement, and stakeholder engagement. The data were analyzed with the support of NVivo software.

**Results:**

The main changes identified involved aligning the incorporation process through standardized evaluation methods, including evidence assessment, economic evaluation, and budget impact analysis. Notably, the analysis deadlines for cancer treatment technologies were shorter in supplementary health (120 days, extendable by 60 days), compared with the public system (180 days, extendable by 90 days). If the supplementary health agency cannot complete the evaluation on time, tacit incorporation occurred. Additionally, technologies recommended by CONITEC were automatically included in the supplementary health framework, ensuring efficient access to effective treatments.

**Conclusions:**

Aligning HTA methods enhances transparency and optimizes analyses. However, certain changes, like mandatory incorporation upon deadline non-fulfillment, were implemented by the legislative branch without comprehensive discussion of their effects on the health system. This lack of dialogue raises concerns about the long-term implications for effective healthcare delivery and stakeholder trust.

